# Doesn’t It All Come Down to Function? How To Correlate VAR2CSA Antibodies with Protection

**DOI:** 10.1128/mSphere.00521-20

**Published:** 2020-06-24

**Authors:** Sedami Gnidehou, Stephanie K. Yanow

**Affiliations:** a Campus Saint-Jean, University of Alberta, Edmonton, Alberta, Canada; b Department of Medical Microbiology and Immunology, University of Alberta, Edmonton, Alberta, Canada; c School of Public Health, University of Alberta, Edmonton, Alberta, Canada; University at Buffalo

**Keywords:** *Plasmodium falciparum*, VAR2CSA antibodies, malaria, men and children, pregnancy

## LETTER

We wish to respond to the article “Going native, or the risk of overreliance on recombinant antigens” (1) by Dr. Lopez-Perez, which questions our findings that antibodies to VAR2CSA can arise outside pregnancy. She asserts that relying on antibody recognition of recombinant proteins can yield misleading data on VAR2CSA antibody responses. We agree; it is not surprising that VAR2CSA may be folded or modified differently when expressed in a variety of cell systems, and this is expected to alter the recognition of the recombinant protein by antibodies. We contend that the most important way to characterize VAR2CSA antibodies is not based exclusively on antigen recognition but on whether those antibodies block the adhesion of infected erythrocytes (IE) to the placental receptor chondroitin sulfate A (CSA). Antibodies with inhibitory activity strongly correlate with protection against pregnancy-associated malaria (PAM) (2–6). The importance of function was further emphasized by Khattab et al. in 2004 (5): “The identification of functional antibodies which appear to inhibit the binding of parasites to CSA in the placenta is crucial to developing a successful vaccination strategy against PAM.”

The most established assay to measure functional antibodies is the inhibition-of-binding assay (IBA). We employed the IBA in all of our studies to show that the VAR2CSA antibodies observed in men and children from Colombia and Brazil were functional (7–9). By virtue of their inhibitory activity in the IBA, these antibodies presumably recognized native VAR2CSA expressed at the surfaces of IE. These findings prompted us to investigate the source of these VAR2CSA antibodies acquired outside pregnancy, which we discovered could be exposure to Plasmodium vivax (8). Sera from men and children with past exposure to P. vivax blocked parasite adhesion. We dissected the underlying mechanism of immune recognition and found that antibodies to P. vivax Duffy binding protein (PvDBP) cross-reacted with VAR2CSA and that both a mouse monoclonal antibody raised against PvDBP and PvDBP-affinity-purified human sera from Colombian men and children had strong blocking activity in the IBA (8). We further mapped the cross-reactive epitope to subdomain 1 (SD1) of PvDBP. Antibodies from Colombian men and children affinity-purified on a synthetic SD1 peptide significantly reduced parasite adhesion to CSA (9).

Collectively, our findings suggest that antibodies to an epitope in PvDBP are functional and inhibit the adhesion of Plasmodium falciparum-infected erythrocytes to CSA. While surprising, heterologous immunity between *Plasmodium* species is not without precedent (10). The Duffy binding-like (DBL) domains of these PvDBP and VAR2CSA proteins are structurally related and likely share epitopes that are evolutionarily conserved. These results point to new epitopes that can be exploited in vaccine design to elicit antibodies to VAR2CSA.

We recognize that our findings challenge the dogma of parity-dependent immunity to PAM. Yet given that current vaccines based on recombinant VAR2CSA domains are hampered by extensive polymorphisms in native alleles and fail to elicit strain-transcending immune responses (11, 12), we hope to stimulate an open scientific dialogue about other, complementary vaccine approaches. Finding a way to protect pregnant women from malaria should be paramount.

## References

[1] Lopez-Perez M 2020 mSphere of Influence: going native, or the risk of overreliance on recombinant antigens. mSphere 5:e00224-20. doi:10.1128/mSphere.00224-20. 32269160 PMC7142302

[2] Fried M, Duffy PE 1998 Maternal malaria and parasite adhesion. J Mol Med (Berl) 76:162–171. doi:10.1007/s001090050205. 9535549

[3] O'Neil-Dunne I, Achur RN, Agbor-Enoh ST, Valiyaveettil M, Naik RS, Ockenhouse CF, Zhou A, Megnekou R, Leke R, Taylor DW, Gowda DC 2001 Gravidity-dependent production of antibodies that inhibit binding of Plasmodium falciparum-infected erythrocytes to placental chondroitin sulfate proteoglycan during pregnancy. Infect Immun 69:7487–7492. doi:10.1128/IAI.69.12.7487-7492.2001. 11705924 PMC98838

[4] Duffy PE, Fried M 2003 Antibodies that inhibit Plasmodium falciparum adhesion to chondroitin sulfate A are associated with increased birth weight and the gestational age of newborns. Infect Immun 71:6620–6623. doi:10.1128/iai.71.11.6620-6623.2003. 14573685 PMC219546

[5] Khattab A, Reinhardt C, Staalsoe T, Fievet N, Kremsner PG, Deloron P, Hviid L, Klinkert MQ 2004 Analysis of IgG with specificity for variant surface antigens expressed by placental Plasmodium falciparum isolates. Malar J 3:21. doi:10.1186/1475-2875-3-21. 15242514 PMC479693

[6] Ndam NT, Denoeud-Ndam L, Doritchamou J, Viwami F, Salanti A, Nielsen MA, Fievet N, Massougbodji A, Luty AJ, Deloron P 2015 Protective antibodies against placental malaria and poor outcomes during pregnancy, Benin. Emerg Infect Dis 21:813–823. doi:10.3201/eid2105.141626. 25898123 PMC4412227

[7] Gnidehou S, Doritchamou J, Arango EM, Cabrera A, Arroyo MI, Kain KC, Ndam NT, Maestre A, Yanow SK 2014 Functional antibodies against VAR2CSA in nonpregnant populations from Colombia exposed to Plasmodium falciparum and Plasmodium vivax. Infect Immun 82:2565–2573. doi:10.1128/IAI.01594-14. 24686068 PMC4019168

[8] Gnidehou S, Mitran CJ, Arango E, Banman S, Mena A, Medawar E, Lima BAS, Doritchamou J, Rajwani J, Jin A, Gavina K, Ntumngia F, Duffy P, Narum D, Ndam NT, Nielsen MA, Salanti A, Kano FS, Carvalho LH, Adams JH, Maestre A, Good MF, Yanow SK 2019 Cross-species immune recognition between Plasmodium vivax Duffy binding protein antibodies and the Plasmodium falciparum surface antigen VAR2CSA. J Infect Dis 219:110–120. doi:10.1093/infdis/jiy467. 30534974 PMC6455908

[9] Mitran CJ, Mena A, Gnidehou S, Banman S, Arango E, Lima BAS, Lugo H, Ganesan A, Salanti A, Mbonye AK, Ntumngia F, Barakat K, Adams JH, Kano FS, Carvalho LH, Maestre AE, Good MF, Yanow SK 2019 Antibodies to cryptic epitopes in distant homologues underpin a mechanism of heterologous immunity between Plasmodium vivax PvDBP and Plasmodium falciparum VAR2CSA. mBio 10:e02343-19. doi:10.1128/mBio.02343-19. 31594821 PMC6786876

[10] Mitran CJ, Yanow SK 2020 The case for exploiting cross-species epitopes in malaria vaccine design. Front Immunol 11:335. doi:10.3389/fimmu.2020.00335. 32174924 PMC7056716

[11] Benavente ED, Oresegun DR, de Sessions PF, Walker EM, Roper C, Dombrowski JG, de Souza RM, Marinho CRF, Sutherland CJ, Hibberd ML, Mohareb F, Baker DA, Clark TG, Campino S 2018 Global genetic diversity of var2csa in Plasmodium falciparum with implications for malaria in pregnancy and vaccine development. Sci Rep 8:15429. doi:10.1038/s41598-018-33767-3. 30337594 PMC6193930

[12] Sirima SB, Richert L, Chene A, Konate AT, Campion C, Dechavanne S, Semblat JP, Benhamouda N, Bahuaud M, Loulergue P, Ouedraogo A, Nebie I, Kabore M, Kargougou D, Barry A, Ouattara SM, Boilet V, Allais F, Roguet G, Havelange N, Lopez-Perez E, Kuppers A, Konate E, Roussillon C, Kante M, Belarbi L, Diarra A, Henry N, Soulama I, Ouedraogo A, Esperou H, Leroy O, Batteux F, Tartour E, Viebig NK, Thiebaut R, Launay O, Gamain B 2020 PRIMVAC vaccine adjuvanted with Alhydrogel or GLA-SE to prevent placental malaria: a first-in-human, randomised, double-blind, placebo-controlled study. Lancet Infect Dis 20:585–597. doi:10.1016/S1473-3099(19)30739-X. 32032566

